# Nucleation and Growth of GaAs on a Carbon Release Layer by Halide Vapor Phase Epitaxy

**DOI:** 10.1021/acsomega.3c07162

**Published:** 2023-11-15

**Authors:** Dennice M. Roberts, Hyunseok Kim, Elisabeth L. McClure, Kuangye Lu, John S. Mangum, Anna K. Braun, Aaron J. Ptak, Kevin L. Schulte, Jeehwan Kim, John Simon

**Affiliations:** † National Renewable Energy Laboratory, Golden, Colorado 80401, United States; ‡ Massachusetts Institute of Technology, Cambridge, Massachusetts 02139, United States; § Colorado School of Mines, Golden, Colorado 80401, United States

## Abstract

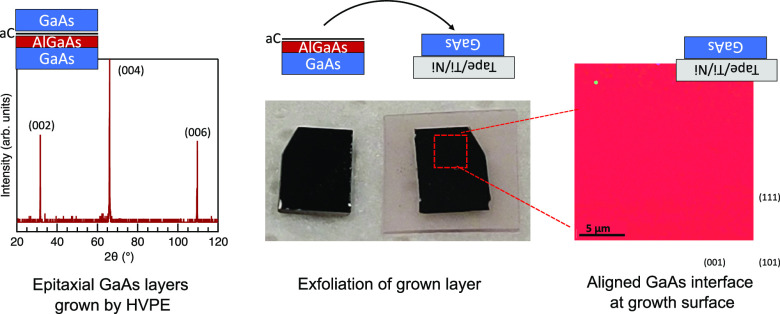

We couple halide vapor phase epitaxy (HVPE) growth of III–V materials with liftoff from an ultrathin carbon release layer to address two significant cost components in III–V device - epitaxial growth and substrate reusability. We investigate nucleation and growth of GaAs layers by HVPE on a thin amorphous carbon layer that can be mechanically exfoliated, leaving the substrate available for reuse. We study nucleation as a function of carbon layer thickness and growth rate and find island-like nucleation. We then study various GaAs growth conditions, including V/III ratio, growth temperature, and growth rate in an effort to minimize film roughness. High growth rates and thicker films lead to drastically smoother surfaces with reduced threading dislocation density. Finally, we grow an initial photovoltaic device on a carbon release layer that has an efficiency of 7.2%. The findings of this work show that HVPE growth is compatible with a carbon release layer and presents a path toward lowering the cost of photovoltaics with high throughput growth and substrate reuse.

## Introduction

Technologies enabling substrate reuse are critical for lowering the cost barrier associated with large area III–V semiconductor devices such as high-efficiency photovoltaics because substrate expenses constitute approximately 30% of the total device cost.^1^ The creation of free-standing devices is also desirable for use in electronics technologies such as strain-modulated transistors, flexible electronics, and power electronics.^2−4^ Substrate reuse technologies are still hindered by issues such as high dislocations, lattice mismatch, and inefficient release processes.^5^ A promising avenue is that of epitaxial liftoff of III–V layers grown on 2D van der Waals (vdW) materials, which provides a fast and simple release mechanism by utilizing the weak vdW bonds in 2D systems as a point of exfoliation.^6−8^ Remote epitaxy has emerged as a 2D-3D substrate reuse technology, in which 3D layers grown on a sufficiently thin vdW material allow epitaxial registry between the grown material and the underlying substrate while still facilitating liftoff.^9,10^ Among other materials, remote epitaxy has been demonstrated for GaN and GaAs through graphene and boron nitride.^9,11^ Recently, it has been determined that strictly vdW materials are not required as long as in-plane bonds are sp^2^ dominant; this enables layers such as amorphous carbon to be deposited directly on the substrate of interest, improving process scalability and bypassing arduous transfer processes.^9,12^

Organometallic vapor phase epitaxy (OMVPE) growth of GaAs-on-GaAs through a thin amorphous carbon interlayer was demonstrated,^12^ but remote epitaxy is yet to be combined with scalable growth techniques that have the potential to lower the cost of the epitaxial growth itself. Growth of III-Vs using halide vapor phase epitaxy (HVPE) may significantly reduce the cost of semiconductor growth due to its high source material utilization, use of elemental group III precursors, and ultrafast growth rates beyond 500 μm/h.^13−15^ Material quality is still maintained at these high growth rates, and HVPE growth of binary, ternary, and quaternary III–V materials with high-quality heterointerfaces results in photovoltaic devices that perform as well as those grown by traditional OMVPE techniques.^16−22^ HVPE growth of III-Vs commonly employs a hydrogen carrier gas, which is likely incompatible with carbon-based interlayers because high flow rates of H_2_ selectively etch graphene.^23^ Studies on graphene interlayers show that exposure to hydrogen process gas results in poor interfaces affecting the quality of the overgrown layer.^9,24^ Instead, the use of a nitrogen carrier gas is another option for HVPE growth that overcomes the 2D layer degradation challenge.^9^ The use of nitrogen may also be an advantage for cost reduction because growth rates of up to 528 μm/h have been reported for GaAs grown by HVPE using a nitrogen carrier gas^15^ and reduce operating cost due to its inert properties.

Here, we study nucleation and growth conditions for GaAs films grown on a thin, amorphous carbon (aC) interlayer, combining the benefits of rapid HVPE growth under nitrogen with efficient substrate reuse. We demonstrate full exfoliation of up to 10 × 10 mm GaAs films, indicating that the underlying aC layer remains sufficiently intact after the HVPE growth process. The grown layer is (001)-oriented and thus maintains a registry to the underlying substrate. We also investigate how growth temperature, growth rate, and V/III ratio affect the resulting morphology of GaAs films grown by HVPE through an aC interlayer and determine planarization conditions that result in smoother films with lower defect densities. Finally, we demonstrate initial photovoltaic devices grown on aC interlayers. The union of HVPE growth and substrate reuse using a weakly bonded interlayer thus presents a pathway toward lower-cost, high-quality optoelectronics.

## Results and Discussion

### 1.1 Nucleation of GaAs Films

**Figure 1:**
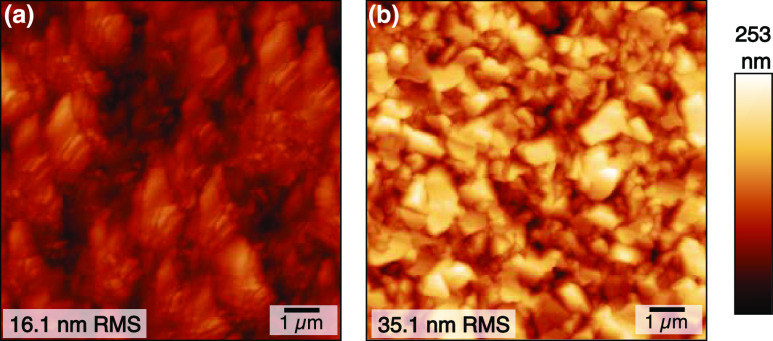
Effect of environmentally exposed carbon interlayers on grown film. (a) AFM scan of GaAs film grown on amorphous carbon layer 2 days after deposition of carbon and subsequent storage in a N_2_ dry box. (b) AFM scan of identical GaAs film grown on the carbon interlayer after the substrate was exposed to the atmosphere within an N_2_ dry box for a week.

We first studied the effects of environmental exposure on the surface of the carbon substrate. The carbon interlayer is grown by an OMVPE and then packaged in an inert atmosphere to transfer for HVPE growth. Figure 1 shows the AFM morphologies of two GaAs layers grown with identical parameters on substrates with different levels of environmental exposure. A GaAs layer was grown on the substrate in Figure 1a after 2 days in a N_2_ environment before transfer to the HVPE reactor and the sample in Figure 1b was grown after the substrate was exposed to an N_2_ dry box environment for one week. Due to the location of the reactors, we were unable to explore samples with less than 2 days of exposure after carbon growth. The sample grown on the more exposed carbon layer is significantly rougher and more faceted. We note that a general trend of very rough or nonplanarized films on older substrates remained constant over many growth attempts, but this work was not able to explore exact chemical changes to the carbon interlayer or sources of degradation. Unless otherwise noted, all samples described in this manuscript are grown within 2 days of carbon substrate growth to minimize these effects as much as possible.

With an initial understanding of sample transfer effects on the carbon substrate, we then studied the effects of aC layer thickness on the nucleation of HVPE-grown GaAs layers. Two different aC layer thicknesses were prepared by varying aC growth time, where the thinner layer was deposited for 5 min and the thicker layer was deposited for 13 min. In both cases, aC layers are deposited on III–V substrates, as described in the Methods. We were not able to determine the exact thickness of the aC layers investigated here due to challenges in measuring layer thicknesses approaching the monolayer limit, but initial studies suggest that the average thickness of aC is around a monolayer for the thinner sample.^12^ Previous studies on graphene interlayers show GaAs quality to be sensitive to the thickness of the 2D interlayer; an overly thick layer suppresses registry with the underlying III–V substrate and an overly thin layer results in incomplete coverage of the substrate and leads to direct bonding between the substrate and film such that the film cannot be exfoliated.^9,10^

**Figure 2:**
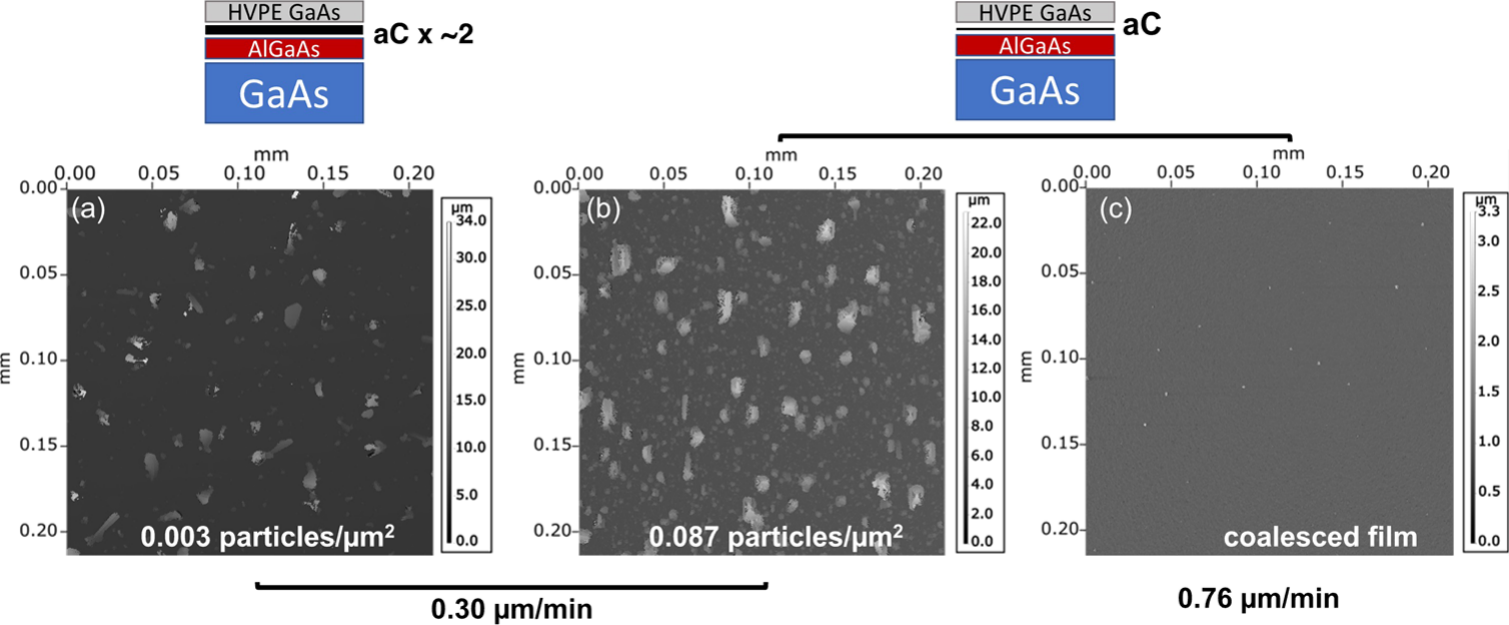
Nucleation of GaAs on an amorphous carbon interlayer. Optical profilometry height map shows GaAs islands for a GaAs growth rate of 0.30 μm/min on (a) a carbon interlayer and (b) a carbon interlayer approximately half as thick. Particle density per area shows an order of magnitude more nucleation sites for the sample grown on the thinner carbon layer. (c) GaAs grown at a higher growth rate of 0.76 μm/min coalesced into cohesive films.

Figure 2 shows the effect of different aC layer thicknesses on the growth of GaAs layers. Here, all films are grown with a V/III ratio of 5 and a growth temperature of 650 °C. We use GaAs growth times leading to a 500 nm thick film on epi-ready substrates as described in the Methods. Figure 2a,b show that for our slowest tested growth rate of 0.30 μm/min, distinct islands form on substrates with both aC thicknesses. This contrasts with smooth films grown on epi-ready GaAs substrates using the same growth conditions; in the absence of a carbon interlayer, we expect the growth of GaAs on a GaAs substrate by HVPE to proceed in a step flow growth mode.^25^Figure 2b shows the density of nucleated islands is significantly higher in the thinner aC layer. Particle analysis (see the Methods) yields nucleation densities of 0.087 and 0.003 islands/μm^2^ for thinner and thicker aC interlayers, respectively. Thick interlayers may shield remote interactions between the substrate and the growing layer,^12^ so increased nucleation density on the thinner aC layer is likely a result of a sufficiently thin interlayer capable of enabling remote epitaxy. Increased island density could also result from nucleation through pinholes of an uncoalesced carbon layer; however, as discussed later and demonstrated in Figure SI.1, coalesced films are fully exfoliated for both aC interlayer thicknesses, leaving intact substrates and films. Spalling marks indicative of pinhole-based selective epitaxy were not observed in this work.^10,26,27^

We also find a growth rate dependence on the film coalescence. Films deposited at 0.30 μm/min, as shown in Figure 2a,b, do not coalesce into planarized films for growth times expected to yield 500 nm thick layers. In contrast, all samples deposited at higher growth rates, but to the same calibrated nominal thickness, result in fully coalesced films, as illustrated in the 0.75 μm/min film in Figure 2c. It is possible that island growth mode is also occurring at higher growth rates prior to film coalescence, but we have not yet been able to confirm this directly.

### 1.2 Exfoliation of Nucleated GaAs Films

**Figure 3:**
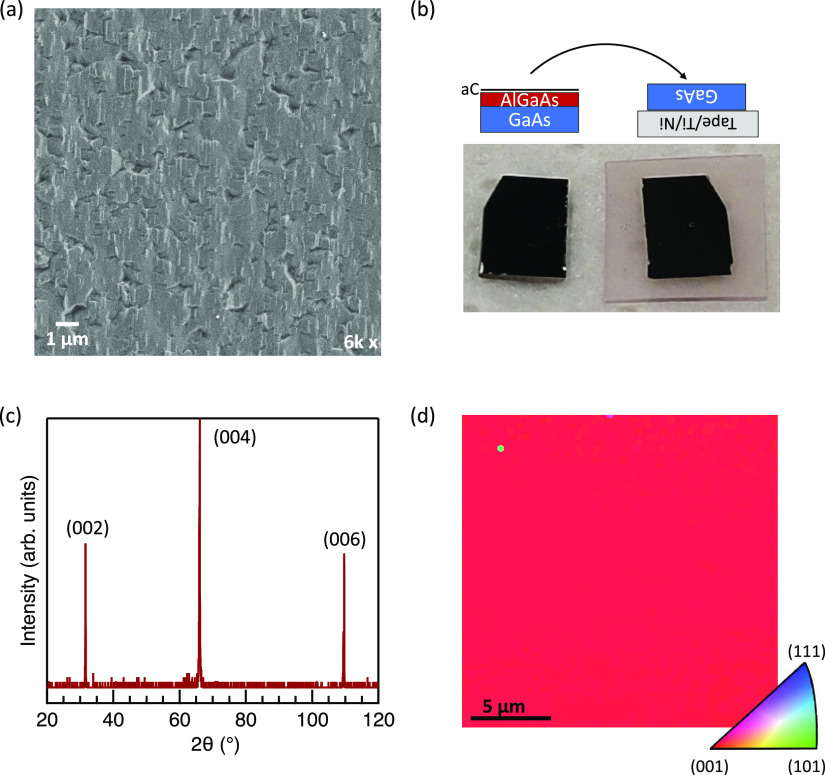
Exfoliation of HVPE-grown GaAs from the substrate at the aC interlayer. (a) SEM of as-grown GaAs film prior to exfoliation. (b) Photograph of the GaAs film after full exfoliation, with the substrate at the left and a grown GaAs layer at the right. The exfoliation process is described further in the Methods. (c) X-ray diffraction of film in (a) prior to exfoliation. (d) EBSD map of the substrate-interfacing side of GaAs film showing (001) as the dominant crystallographic orientation.

A primary goal of this work is to demonstrate HVPE as a viable III–V growth method on carbon interlayers for substrate reuse. As discussed before, a N_2_ process gas is used to offset the potential for etching of the aC interlayer by H_2_; however, hydride-based precursors are still used as part of the HVPE process and may lead to etching of the aC layer. An incomplete aC interlayer will impact the ability to exfoliate layers from the parent substrate. As such, we test exfoliation of a fully coalesced GaAs film to determine if the carbon layer remains intact after film growth. Figure 3a shows an SEM micrograph of an as-deposited 250 nm-thick film grown using the growth conditions of the sample in Figure 2c prior to exfoliation. Films are exfoliated using a nickel stressor layer as described in the Experimental Methods section. We show the separated film and substrate postexfoliation in Figure 3b. Following exfoliation, the GaAs layer is fully intact as a free-standing film, implying the aC layer remains sufficiently intact prior to and during film growth and is not substantially etched or damaged by the HVPE-specific chemistries present in the reactor. The fully freestanding film also suggests that aC deposition is uniform over the area of the III–V substrate, as large changes in carbon interlayer thickness or would result in poor crystallinity or incomplete exfoliation, respectively.^9^

The structure is investigated at the front and back surfaces of the film to determine the registry of the grown layer with the substrate. X-ray diffraction, shown in Figure 3c, shows the registry of the layer to the substrate because the film is fully oriented in the (001) direction with no peaks associated with non-(001) family planes. We performed EBSD to investigate local grain orientation at the nucleating surface by measuring the bottom of the film following exfoliation. Figure 3d shows EBSD patterns of the freestanding GaAs film at the initial growth interface with the aC interlayer. The GaAs film is fully (001)-oriented, consistent with the X-ray diffraction data shown in Figure 3c. There is also no evidence of in-plane rotation from EBSD pole figures (not shown). Previous work showed that OMVPE-grown GaAs on a graphene/GaAs substrate were smoother and displayed lower polycrystallinity when the interlayer thickness decreased from a bilayer to a monolayer of graphene.^9,11^ EBSD results are almost uniformly (001)-oriented and more closely resemble the results for growth on a monolayer of graphene than growth on a bilayer.^11^ This further suggests that the aC interlayer is sufficiently thin to allow interaction between the substrate and growing layer as a thick, uninterrupted layer of carbon results in polycrystalline growth.^9^ While a demonstration of regrowth and thus substrate reuse was outside the scope of this work, examples of substrate reuse through an exfoliated GaAs-on-aC interlayer are shown in a previous study.^12^

### 1.3 Optimization of GaAs Growth Conditions

**Figure 4:**
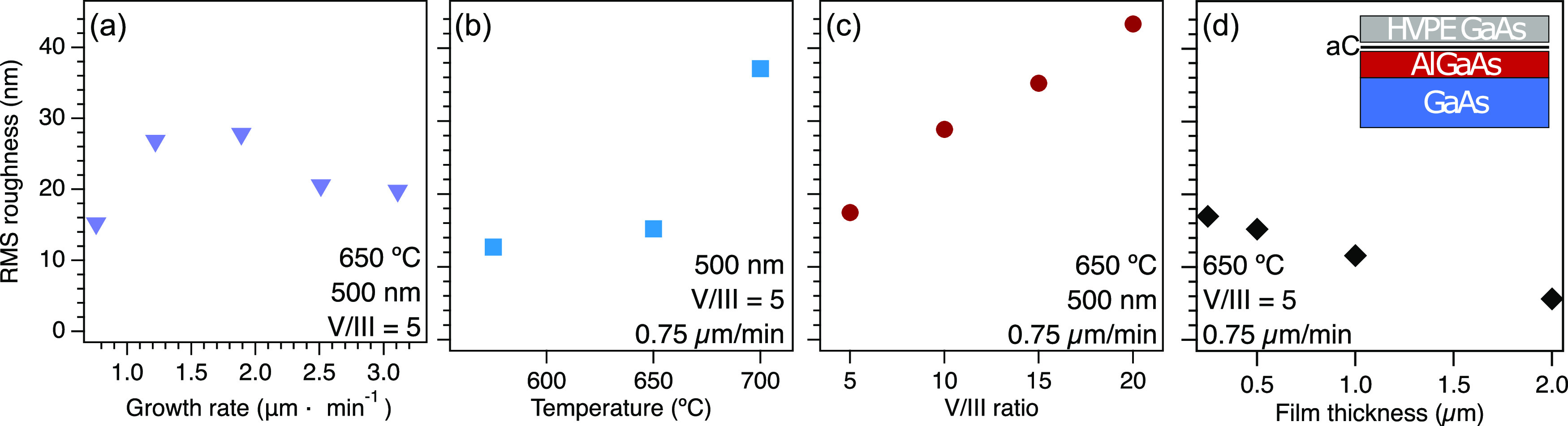
Optimization of GaAs growth parameters on aC-coated III–V substrates. AFM-determined roughness of GaAs films nucleated at various conditions on an amorphous carbon interlayer using different (a) growth rates, (b) growth temperatures, (c) V/III ratios, and (d) film thicknesses. Inset: growth schematic for all growths described.

Initial measurements of films show a high surface roughness unlikely to accommodate high-quality device growth;^28^ further optimization of growth conditions was thus required to minimize this parameter. We consider the effect of the V/III ratio, temperature, and growth rate on films with an approximate thickness of 500 nm. The inset of Figure 4 shows the growth schematic for these films. Figure 4a–c show roughness as determined by atomic force microscopy over a 100 μm^2^ scan area as a function of growth rate, growth temperature, and V/III ratio, respectively. All films are single crystals, fully coalesced, and (001) oriented with no detectable competition from other crystallographic orientations (see Figure SI.2). We note that all samples studied here were (001)-oriented GaAs like that of the sample demonstrated in Figure 3. Figure 4a shows the roughness of GaAs films at growth rates between 0.75 and 3.11 μm/min where the growth temperature is held constant at 650 °C and V/III = 5. Roughness increases for growth rates above 0.75 μm/min before dropping again at growth rates above 2.0 μm/min. Figure 4b shows that the roughness of films with varied growth temperature and a constant growth rate of 0.75 μm/min and V/III = 5 is the lowest for a growth temperature of 575 °C; however, we choose to further optimize films at a growth temperature of 650 °C, despite the very small increase in roughness over the sample grown at 575 °C to accommodate the temperature where we have optimized most of our materials and devices.^19^

Figure 4c shows an increase in roughness with an increasing V/III ratio for films grown at a constant temperature of 650 °C and a growth rate of 0.75 μm/min. The lower V/III could lead to a lower density of nucleation sites that result in a lower number of boundaries at the coalescence front than might be generated at a higher V/III ratio, resulting in smoother films. Finally, we investigate changes to film morphology as a function of film thickness to understand the smoothing of facets visible in thinner films such as the film shown in Figure 3a. Figure 4d shows the RMS roughness of films as a function of film thickness using growth conditions where V/III = 5, growth temperature is 650 °C, and growth rate is 0.75 μm/min. For growth rates high enough to result in planar films, film roughness decreases with the GaAs layer thickness. The RMS roughness of the 2-μm-thick film scanned over a 10 × 10 μm area is 5.1 nm.

**Figure 5:**
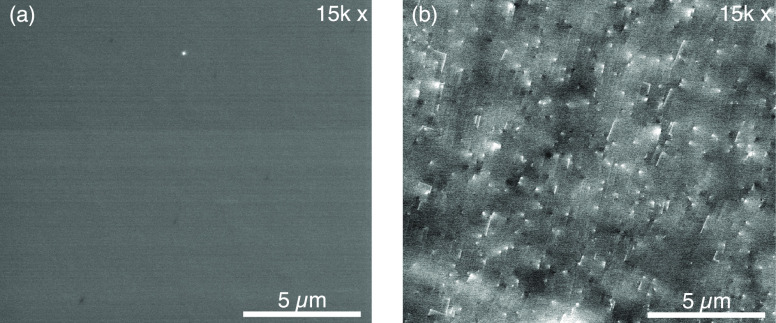
Effect of growth optimization on the surface morphology, as described in text. (a) SEM of a 2-μm-thick film using conditions optimized by the parametric studies described in Figure 4. (b) Representative ECCI of the film in (a) showing threading dislocations and stacking faults.

We used the findings of the parametric studies to optimize the growth of a GaAs layer on aC. We achieved a further reduction of film roughness from the films demonstrated above using a low V/III of 5, a growth temperature of 650 °C and a growth rate of about 4 μm/min, which has been increased via an increased flow of nitrogen through the center tube to 4000 sccm. Figure 5a shows an SEM micrograph of the surface of this sample. Reflectance modeling confirms this film to be 2 μm thick, and AFM shows a surface roughness of 2.2 nm, the lowest found in this work. By comparison, polished GaAs substrates have a roughness on the order of 0.4 nm.^29^ The conditions used here are consistent with planarization conditions optimized in recent work studying HVPE growth on roughened substrates under hydrogen gas.^29^ This work shows that similar planarization results occur under nitrogen.

Low dislocation density is critical for achieving high-performance devices because dislocations serve as recombination sites for the generated carriers. Figure 5b shows an electron channeling contrast imaging (ECCI) measurement of the threading dislocation density of the film in Figure 5a. We find an average dislocation density of 2.8 × 10^8^ cm^–2^ for measurements averaged over five 250 μm^2^ regions. This defect density is a significant improvement from the dislocation density in the unoptimized films from Figure 4 (>5 × 10^8^ cm^–2^ and thus above the detector threshold) but is high enough to adversely impact device performance. These dislocations possibly derive from the merging of islands after nucleation, but further investigation is warranted.^30^ Another potential route for improvement is in the quality of the amorphous carbon layer. Our observations in Figure 1 suggest that similar issues with air exposure exist in this work because nominally identical GaAs layers grown on air-exposed substrates show more macroscopically defective regions than less-exposed substrates. Recent work shows that the quality and source of the carbon interlayer in these GaAs-carbon-GaAs systems can affect the quality of the grown layer,^9^ and we, therefore, suspect that further optimization of the aC surface could reduce the defect density of our GaAs layers by promoting more uniform film growth.

### 1.4 Initial Device Demonstration

**Figure 6:**
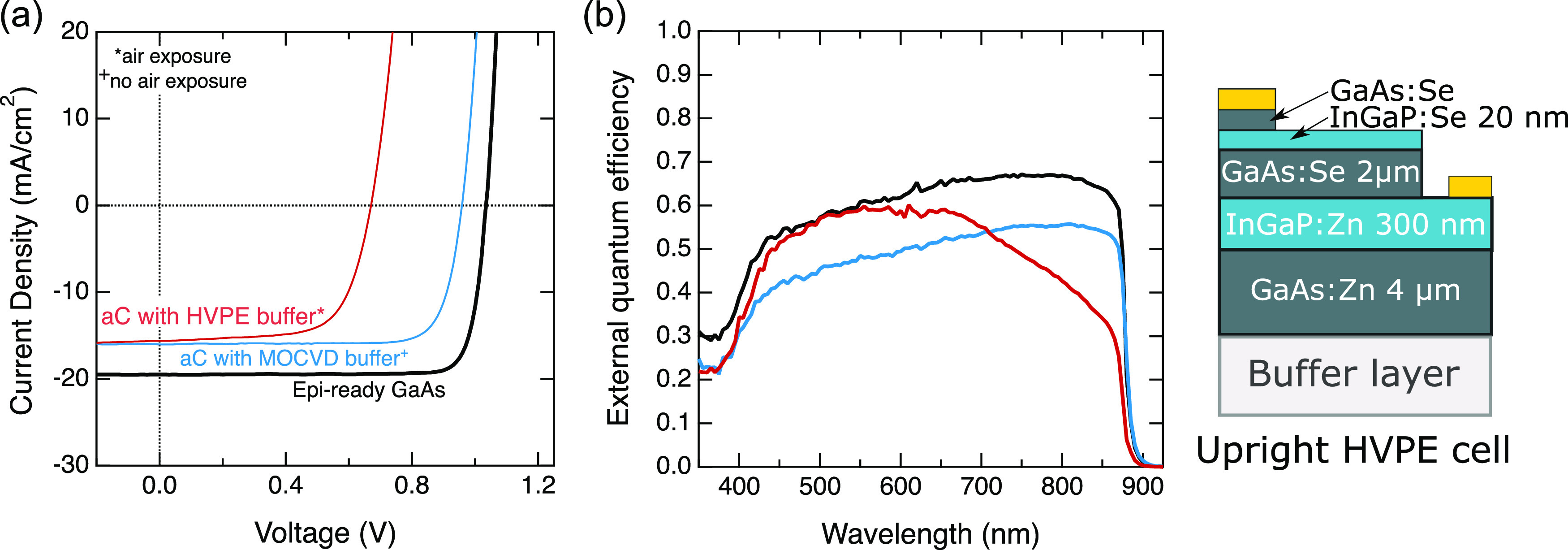
Initial demonstration of HVPE-grown solar cells on an aC interlayer (a) current density–voltage curves of an upright HVPE cell grown on a GaAs buffer grown by HVPE (red) and a reference GaAs cell on an epi-ready GaAs wafer (black). (b) Quantum efficiency curves of devices described in (a). The schematic on the right shows the device structure; layer thickness is not to scale. Devices do not have an antireflection coating.

We grew an upright, rear heterojunction GaAs solar cell on a GaAs substrate with an aC interlayer to show an initial demonstration of device performance. Figure 6 shows the performance of the upright GaAs solar cells and the schematic of the full device layer structure. Neither cell has an anti-reflection coating. The black traces represent the results of a control cell grown on an epi-ready GaAs wafer with no aC interlayer that serves as a performance baseline. Red traces represent a cell grown on an aC substrate with a ∼3 μm thick GaAs buffer layer grown using the planarization conditions from Figure 5. We determine cell efficiencies from the current density–voltage traces, as shown in Figure 6a. The measured conversion efficiencies are and 7.2% for the control cell and carbon interlayer cell, respectively. Table 1 outlines the full performance metrics of the two cells. It is well-known that dislocations lead to a reduction in the short circuit current density (*J*_SC_) and open circuit voltage (*V*_OC_) in solar cells. The carbon interlayer cell has an efficiency reduction relative to the control that suggests the presence of TDD on the order of 10^8^ cm^–2^ based on previous studies of defective cells, in agreement with our ECCI results.^30^Figure 6b shows the external quantum efficiency (EQE) for each of the cells described here. In the carbon interlayer cell, a significant falloff in EQE at higher wavelengths implies rear surface recombination that likely results from the defects localized at the rear of the cell near the buffer layer.

**Table 1:** Performance Metrics for Uncoated Cells Described in Figure 6

sample	*J*_**SC**_ (mA/cm^2^)	*V*_**OC**_**(V)**	fill Factor (%)	efficiency (%)
baseline, epi-ready GaAs	19.79	1.05	82.67	17.1
aC interlayer, HVPE buffer	15.37	0.69	67.99	7.2

Unfortunately, these cells were unable to be grown immediately after the growth of the aC substrates, so this study is unable to decouple the detrimental effects of coalescence and carbon degradation. In the ideal case, the carbon layer and subsequent device would be grown either in-line or without breaking vacuum. As an example, Figure SI.3 shows the same cell structure grown by HVPE on an OMVPE-grown buffer wherein no break in vacuum occurred between the growth of the aC layer and the buffer layer. This comparison provides a picture of what is possible for an HVPE-grown cell on these reusable templates; other explanations of cell performance indicated by this comparison are the subject of a future study. At this time, we are unable to determine if improved performance is due to superior nucleation by OMVPE, reduced carbon layer degradation, or some combination; further study is needed.

## 2 Conclusions

This study presents the successful nucleation of GaAs on III–V substrates through a thin carbon interlayer. The interlayer material here is amenable to wafer-scale deposition; the exfoliation method is relatively rapid, and the growth method described here is capable of producing III–V devices at high throughput. We show single crystal films that fully exfoliate from the substrate, demonstrating that these techniques are possible in tandem. We further investigate the effect of varied growth conditions on film quality, namely, film roughness and defect density, and find that film quality is significantly improved for growth conditions with low V/III ratios and high growth rates at or above around 0.75 μm/min. Planarization techniques drive film morphologies toward smooth surfaces better suited for device growth. Initial device growth yields a cell efficiency of 7.2% (without antireflection coating) for HVPE-grown single junction upright GaAs cells; device quality can likely improve by limiting air exposure to the carbon layer prior to transfer to the HVPE reactor.

## 3 Experimental Methods

### 3.1 Growth of III–V/aC Substrates

Approximately 500 nm thick AlGaAs layers were deposited by OMVPE on a GaAs substrate with a 6° offcut toward (111)A. AlGaAs were chosen as they are more thermally robust than GaAs and are thus a more suitable candidate for the subsequent carbon deposition process. An amorphous carbon layer was then deposited at MIT on the AlGaAs substrate by an OMVPE after heating to around 700 °C; flowing toluene and nitrogen are used as a carbon precursor and a carrier gas, respectively. More details on the substrate preparation process can be found in ref (12). After growth, the aC substrates were vacuum sealed and sent to NREL for deposition in the HVPE reactor. Upon receipt, samples were immediately unloaded and transferred to the HVPE reactor for growth. These efforts aim to reduce air exposure as much as possible in the absence of fully air-free transfer options.

### 3.2 HVPE Growth of Layers and Devices

GaAs films were grown in a custom two-growth-chamber HVPE reactor using a nitrogen process gas described in detail elsewhere.^13,15^ Boats containing elemental group III precursors were held at a constant temperature of 800 °C and the growth zone temperature was varied between 575 and 750 °C as noted in the text. Thickness calibration samples were prepared on epi-ready GaAs substrates with a 6° offcut toward the (111)A face using growth conditions identical to those used for growth on aC interlayers. Growth rates were varied by modulating the flow of the nitrogen carrier gas that delivers uncracked hydrides to the substrate surface as described in previous studies.^15,20^ For devices, the n-type dopant is selenium and is provided by a flow of H_2_Se; the p-type dopant is zinc and is provided by a diethylzinc source.

### 3.3 Exfoliation Method

The GaAs film grown on aC was exfoliated by using the following process. First, a 35 nm-thick Ti layer is deposited by electron-beam evaporation as an adhesion layer. Next, a high-stress Ni stressor layer is deposited by DC sputtering with a thickness of 3–7 μm. A thermally releasable tape is then attached on the Ni, and the entire stack of tape/Ni/Ti/GaAs layers is manually exfoliated at the aC interface by mechanical peeling. This method is described in more detail in Reference.^12^

### 3.4 Material Characterization

Nucleation densities for GaAs islands were calculated from optical profiles captured by a Keyence profilometer. Data processing was performed in ImageJ using the MorphoLibJ package. X-ray diffraction was collected using a Panalytical X’Pert Pro instrument in a symmetric scan mode. The growth rate was calibrated by first growing a GaInP layer on an epi-ready GaAs substrate and then growing a GaAs layer under the same growth conditions as those used on aC; the top GaAs layer was partially masked and selectively etched, and layer height was determined by contact profilometry.

Electron backscatter diffraction (EBSD) patterns are collected using a Zeiss Merlin high-resolution scanning electron microscopy (SEM). A beam acceleration voltage of 15 kV and a current of 3 nA are used for the EBSD mapping. Atomic force microscopy (AFM) was collected using a Nanosurf EasyScan 2 collected over a 100 μm^2^ scan area with 3 s of collection time per line. Average roughness was characterized by using instrument software analysis over the full scan area following background correction. Electron channeling contrast imaging (ECCI) was conducted on an FEI Nova NanoSEM 630 operating at 25 kV accelerating voltage and 3.2 nA beam current and defect densities were calculated by counting the number of defects within a total imaging area of approximately 1250 μm^2^. SEM was performed on the same instrument operating at a 3 kV accelerating voltage and 0.64 nA beam current.

### 3.5 Cell Measurements

Devices are processed using standard photolithography techniques, and Au metal is electroplated for both front and rear contacts. Light and dark current density as a function of voltage (J-V) is collected on an XT-10 continuous wave solar simulator with a xenon arc lamp under a simulated AM1.5G spectrum. Light intensity is calibrated against a GaAs reference cell to simulate 1-sun conditions. Quantum efficiency was measured using a home-built system that contains a reflectance diode; reflectance spectra were used to determine the layer thickness. Cells measured here do not have an anti-reflective coating and have not been certified by an independent laboratory.

## Supporting Information Available

Image of exfoliated films; wide-range X-ray diffraction scans for grown GaAs films; and performance data for a reference solar cell structure grown entirely by MOCVD (PDF)
