# Supplementary material for: Nucleation and Growth of GaAs on a Carbon Release Layer by Halide Vapor Phase Epitaxy
Source: ACS Omega. 2023 Nov 15;8(47):45088–95. doi: 10.1021/acsomega.3c07162 (PMC10688164; doi:10.1021/acsomega.3c07162)
Supplement: Supplementary file 1 — ao3c07162_si_001.pdf [file ao3c07162_si_001.pdf]

Supporting information for

Nucleation and growth of GaAs on a carbon release layer by halide vapor phase epitaxy

Dennice M. Roberts[1*], Hyunseok Kim[2], Elisabeth L. McClure[1], Kuangye Lu[2], John S. Mangum[1], Anna K. Braun[3], Aaron J. Ptak[1], Kevin L. Schulte[1], Jeehwan Kim[2], John Simon[1*]

[1]National Renewable Energy Laboratory, Golden, Colorado 80401
[2]Massachusetts Institute of Technology, Cambridge, MA 02139
[3]Colorado School of Mines, Golden, Colorado 80401

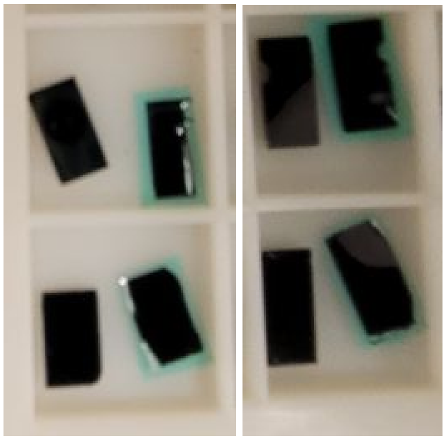

Figure SI.1 Exfoliation of GaAs thin films on the two carbon layer thicknesses discussed in Figure 1. The left piece in each quadrant is the substrate and the right piece of each quadrant is the fully exfoliated films on release layers (blue). Top left: thinner carbon layer, 250 nm GaAs film. Top right: thicker carbon layer, 250 nm GaAs film. Bottom left: thinner carbon layer, 500 nm GaAs film. Bottom right: thicker carbon layer, 500 nm GaAs film.

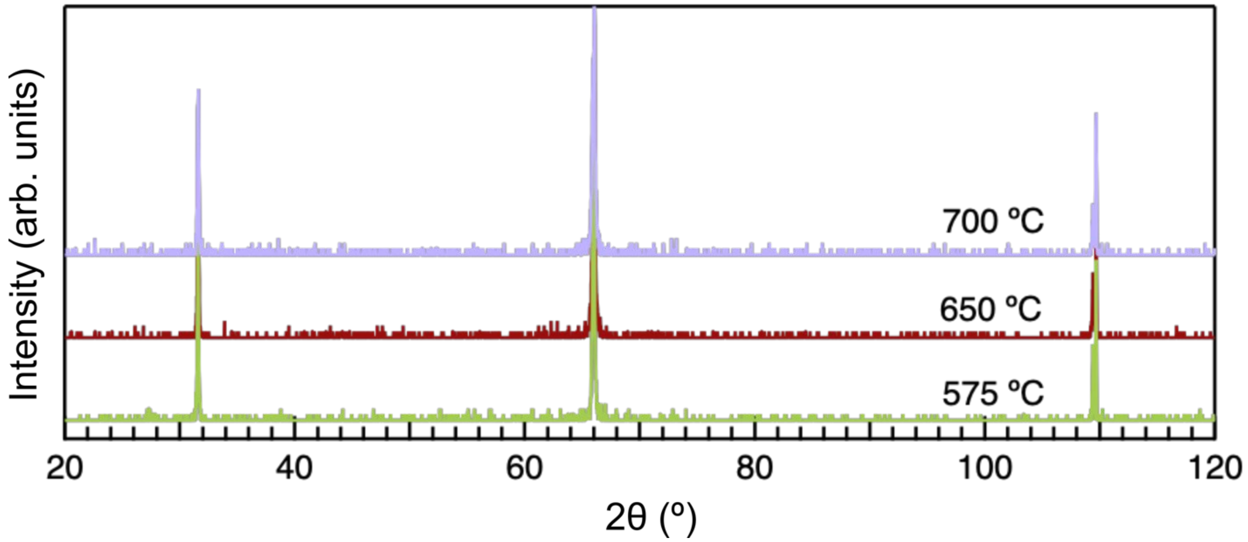

Fig SI.2 X-ray diffraction patterns of GaAs films at three different growth temperatures.

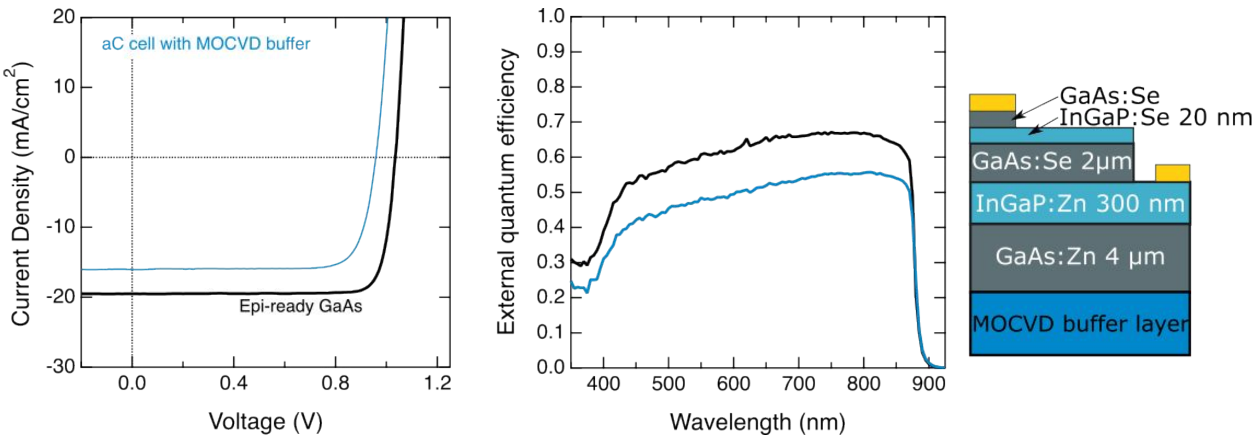

Sample	J_{SC} (mA/cm²)	V_{OC} (V)	Fill Factor (%)	Efficiency (%)
Baseline, epi-ready GaAs	19.79	1.05	82.67	17.1
aC interlayer, HVPE buffer	15.37	0.69	67.99	7.2
aC interlayer, MOCVD buffer	16.02	0.96	80.25	12.3

Fig SI.3 HVPE-grown GaAs solar cell with an identical structure to the cell in Fig 6 but with an MOCVD-grown GaAs buffer layer instead of an HVPE-grown buffer layer. The MOCVD-grown buffer is deposited immediately after the aC interlayer without breaking vacuum. Performance of all cells described in this work is noted in the table.
